# An intersectional approach for the effects of school bullying & cyberbullying on beliefs about the future among Latine LGBTQ+ youth in the United States

**DOI:** 10.1111/jora.70247

**Published:** 2026-08-10

**Authors:** Aldo M. Barrita, Roberto L. Abreu, David G. Zelaya, Joshua G. Parmenter, Nathan Williams, Ryan J. Watson

**Affiliations:** ^1^ Department of Psychology Michigan State University East Lansing Michigan USA; ^2^ Department of Psychology University of Florida Gainesville Florida USA; ^3^ Department of Behavioral and Social Sciences Brown University School of Public Health Providence Rhode Island USA; ^4^ School of Counseling and Counseling Psychology Arizona State University Tempe Arizona USA; ^5^ Department of Human Development and Family Sciences University of Connecticut Mansfield Connecticut USA

**Keywords:** beliefs about future, bullying, intersectionality, Latine, LGBTQ+

## Abstract

Latine lesbian, gay, bisexual, transgender, and queer (LGBTQ+) youth in the United States (U.S.) experience compounded forms of oppression that increase vulnerability to both school‐based and cyberbullying. Guided by the Minority Stress Theory (MST) and the Temporal Intersectional Minority Stress Model (TIMSM), this study examined how intersectional bullying relates to future outlook among Latine LGBTQ+ youth, and whether internalized stigma, depression, and caregiver acceptance mediate or moderate these associations. Data were drawn from a national U.S. sample of 1772 Latine LGBTQ+ adolescents aged 13–18. Participants completed validated measures assessing intersectional school and cyberbullying, LGBTQ+ internalized stigma, depression‐anxiety, caregiver acceptance, and beliefs about the future. Serial indirect‐effects and moderation analyses were conducted. Both school‐based bullying and cyberbullying were indirectly associated with more negative beliefs about the future through increased LGBTQ+ internalized stigma and depressive/anxiety symptoms. Cyberbullying demonstrated slightly stronger links to internalized stigma, whereas school bullying was more closely tied to depressive/anxiety outcomes. LGBTQ+ caregiver acceptance at high levels significantly buffered the effects of both forms of bullying on internalized stigma and depression. Findings underscore the cumulative impact of intersectional bullying on Latine LGBTQ+ youths' mental health and future outlook within the U.S. sociocultural context. Culturally responsive interventions that strengthen caregiver acceptance, promote affirming school climates, and address digital victimization are essential for fostering resilience and hope among Latine LGBTQ+ youth in the U.S.

## INTRODUCTION

Lesbian, gay, bisexual, transgender, and queer (LGBTQ+) youth in the United States face unique stressors rooted in cisheterosexism that undermine their psychological well‐being (Schnarrs et al., 2019). One of the most pervasive of these stressors is bias‐based bullying, which is harassment or victimization targeting individuals because of their actual or perceived identities (Jones et al., 2018). Compared to their heterosexual and cisgender peers, LGBTQ+ youth in U.S.‐based samples are disproportionately exposed to bullying both in school and online (Abreu & Kenny, 2018; Angoff & Barnhart, 2021). For racialized LGBTQ+ youth, such as Latine LGBTQ+, these experiences often extend beyond sexual and gender identity, reflecting multiple intersecting systems of oppression, such as racism. Latine LGBTQ+ youth in the U.S. experience compounded forms of bias‐based bullying (Abreu et al., 2023, 2026; Barrita, Abreu, et al., 2025; Barrita et al., 2026; Barrita, Parmenter, et al., 2025; Kosciw et al., 2018); Intersectional bullying, which includes experiences of racism and cisheterosexism, has been linked to poorer mental health outcomes and reduced engagement in health‐promoting behaviors, such as lower willingness to consider preventive care for sexually transmitted infections (Abreu et al., 2025). Cyberbullying, which is harassment or mistreatment experienced through digital or electronic platforms, represents a particularly salient form of victimization for LGBTQ+ youth (Abreu & Kenny, 2018). Importantly, adolescence (ages 13–18) represents a developmental period characterized by identity formation, heightened sensitivity to peer evaluation, and increasing future orientation, all of which may amplify the impact of bullying and discrimination on both psychological functioning and expectations about the future (Hirsch et al., 2017). For Latine LGBTQ+ adolescents in the U.S., these developmental processes unfold within broader sociopolitical and cultural contexts that shape experiences of safety and identity affirmation across school and online environments. These broader structural contexts may also influence how bullying is experienced and how adolescents develop expectations about their future.

Repeated exposure to intersectional oppression, among Latine LGBTQ+ in the United States, is associated with heightened psychological distress, internalized stigma, and negative coping strategies (Abreu et al., 2025, 2026; Barrita, Abreu, et al., 2025; Barrita et al., 2026; Barrita, Parmenter, et al., 2025), all factors that may shape how youth view their futures and possibilities for thriving as LGBTQ+ individuals. Given these risks, it is important to examine how bullying experiences, both in‐person and online, are associated with mental health and future‐oriented beliefs among Latine LGBTQ+ youth living in the United States. This study addresses this gap using an intersectional approach by examining the links between bullying (school and cyber), psychological distress, and beliefs about the future among Latine LGBTQ+ youth in the United States, a population at the intersection of multiple marginalized identities, yet often overlooked in research. In the present study, intersectionality is conceptualized as the co‐occurrence of multiple minoritized identities (e.g., racial/ethnic and LGBTQ+ identities) that shape exposure to overlapping forms of bias‐based bullying across contexts, rather than as a comparative framework across identity groups. This approach aligns with prior research using composite measures of intersectional victimization to capture cumulative exposure to identity‐based mistreatment (Abreu et al., 2025, 2026; Barrita et al., 2023; Barrita, Hixson, et al., 2024; Parmenter & Barrita, 2026a, 2026b).

### Minority stress model and internalized LGBTQ stigma

Persistent exposure to discrimination and victimization can shape how Latine LGBTQ+ youth feel about their sense of self. Minority Stress Theory (MST; Brooks, 1981; Meyer, 2003) provides a useful framework for understanding how these experiences are associated with mental health outcomes among Latine LGBTQ+ youth in the United States. MST posits that distal (i.e., external stressors such as discrimination, microaggressions, and bullying) and proximal (i.e., internal stressors, such as internalized stigma or shame about one's LGBTQ+ identity) stressors are linked to mental health disparities. Further, experiences of discrimination and bullying (i.e., distal stressors) can become internalized (i.e., proximal stressors) and are associated with psychological outcomes for LGBTQ+ populations (Barrita, Abreu, et al., 2025; Barrita et al., 2026; Parmenter & Barrita, 2026a). MST provides a valuable lens for understanding stress among LGBTQ+ youth (Hatzenbuehler & Pachankis, 2016), yet it has been critiqued for more limited attention to how intersecting forms of oppression jointly shape mental health disparities among individuals with multiple minoritized identities.

To address these limitations, the Temporal Intersectional Minority Stress Model (TIMSM; Rivas‐Koehl et al., 2023) extends MST by emphasizing how intersecting identities and stressors unfold over time and across contexts, capturing both the cumulative and dynamic nature of oppression experienced by individuals with multiple minoritized identities. TIMSM further highlights that stress processes are not strictly linear but may be recursive, such that distal stressors (e.g., bullying) and proximal stressors (e.g., internalized stigma, depressive symptoms) can reinforce one another over time and across ecological contexts (Rivas‐Koehl et al., 2023). Although the present study uses cross‐sectional data and cannot directly model temporal or recursive processes, the conceptual model is informed by these theoretical assumptions and examines patterns of association that are consistent with temporally ordered stress processes. TIMSM also emphasizes the role of protective factors and social support system**s**, such as family acceptance, community connectedness, and cultural resilience, in buffering the effects of distal and proximal stressors on psychological well‐being.

Researchers continue to document how experiences of discrimination and bullying have deleterious effects on the lives of LGBTQ+ youth (Abreu & Kenny, 2018; Angoff & Barnhart, 2021), with even more pronounced disparities among Latine LGBTQ+ people (Barrita, Parmenter, et al., 2025; Velez et al., 2015). Moreover, experiences of bullying contribute to heightened internalized shame and stigma, reinforcing negative self‐perceptions and identity‐based devaluation among minoritized youth (Abreu & Kenny, 2018; Gower et al., 2018). Similar effects have been seen among Latine LGBTQ+ youth living in the United States experiencing intersectional bullying and cyberbullying, with increased LGBTQ+ internalized stigma and adverse mental health outcomes (Abreu et al., 2025, 2026; Rosales et al., 2023; Toomey et al., 2018). Notably, these studies often capture intersectionality through cumulative exposure to multiple identity‐based stressors rather than isolating single identity domains, reflecting the complexity of lived experiences among multiply marginalized youth. Understanding how intersectional forms of bullying are associated with mental health is integral for addressing mental health disparities among Latine LGBTQ+ in the United States through clinical practice and outreach. However, little is known about how distal (e.g., intersectional bullying in‐person or online) and proximal stressors (e.g., internalized stigma) may also relate to perceptions and beliefs about the future for Latine LGBTQ+ youth in the United States.

### Bullying for Latine and LGBTQ+ youth

Latine LGBTQ+ youth in the United States encounter various forms of health disparities as a result of living at the nexus of multiple forms of oppression, such as racism and cisheterosexism (Rosales et al., 2023). The mechanisms through which these systems manifest are discrimination, victimization, and violence, which have been associated with numerous deleterious health outcomes (Abreu & Kenny, 2018; Angoff & Barnhart, 2021; Jones et al., 2018). A relevant, yet understudied, manifestation of these intersecting systems of oppression is bullying among Latine LGBTQ+ youth living in the U.S. (Abreu et al., 2025). Bullying can be described as intentional and repeated forms of aggression toward an individual, which can be experienced through direct and indirect forms as a result of a power imbalance between the perpetrator (bully) and target (Angoff & Barnhart, 2021; Jones et al., 2018). Direct forms of bullying include overt forms of aggression, such as physical violence, and indirect forms of bullying that are more relational in nature, such as spreading rumors to harm the individual (Angoff & Barnhart, 2021). Within youth, bullying has been conceptualized as occurring between peers within school settings, within familial relationships, intimate relationships, and even anonymous users in the online world. Because adolescents spend a substantial portion of their time in school, these settings represent a primary context in which identity‐based victimization shapes perceptions of safety, belonging, and peer relationships among Latine LGBTQ+ youth in the United States. For these youth, school safety encompasses more than physical protection; it also reflects whether schools are affirming, free from identity‐based harassment, and supported by inclusive educators, peers, and institutional policies. Consequently, bullying may signal not only interpersonal peer aggression but also broader deficiencies in school climate that shape identity development and psychological well‐being.

Prior research examining singular axes of oppression and associated experiences of bullying shows that between 15 and 30% of youth are subject to bullying in their lifetime (Modecki et al., 2014), with rates varying based on racial/ethnic background, sexual orientation, gender identity, and other sociocultural minoritized identities. Broadly, the Latine community is one of the largest ethnic groups and represents a high proportion of students enrolled in K‐12 education. Within the current U.S. sociopolitical climate, Latine youth are experiencing immigration‐related bullying, which may be compounded by other forms of discrimination and acculturative stress (Barrita, Carbajal, et al., 2024; Brietzke & Perreira, 2017). For Latine LGBTQ+ youth in the United States, these experiences may intersect with sexual‐ and gender‐based stigma, contributing to school environments in which safety and belonging are not experienced equally across students.

Prior research examining the experiences of bullying within LGBTQ+ youth in the United States is robust and clear, underscoring that they encounter significantly higher levels of bullying victimization than their heterosexual counterparts (Abreu & Kenny, 2018; Angoff & Barnhart, 2021; Jones et al., 2018; Modecki et al., 2014). These experiences of bullying victimization have been associated with numerous negative health disparities, such as higher rates of substance use, mood disorders, suicidal ideation, and suicide attempts (Abreu et al., 2023; Arcadepani et al., 2021; Barrita et al., 2026; Garcia‐Perez, 2020; Giano et al., 2021). Importantly, for Latine LGBTQ+ youth in the United States, these forms of victimization are not experienced in isolation. Rather, bullying may simultaneously target multiple aspects of identity (e.g., race/ethnicity, sexual identity, gender expression, immigrant identity), reflecting the co‐occurrence of intersecting stigmatized identities within a single experience. Research examining and documenting the experiences of Latine LGBTQ+ youth and bullying remains nascent, likely due to intersectionality invisibility (Purdie‐Vaughns & Eibach, 2008). Yet, evidence has shown that Latine LGBTQ+ youth encounter higher levels of bullying compared to their white and heterosexual counterparts (Abreu et al., 2026). Barrita, Parmenter, and colleagues (Barrita, Parmenter, et al., 2025) found that intersectional school‐bullying experiences among Latine LGBTQ+ youth were positively associated with psychological distress and internalization in a large national sample. In the present study, intersectional bullying is operationalized as cumulative exposure to bullying across multiple identity domains (e.g., race/ethnicity, sexual identity, gender, immigrant identity), consistent with prior work using composite indices to capture the breadth of identity‐based victimization (Abreu et al., 2025; Barrita, Abrue, et al., 2025, 2026). While this approach does not isolate the unique effects of specific identity‐based experiences, it reflects the reality that youth may experience overlapping and simultaneous forms of bias‐based bullying in their daily lives.

With the rise of technology and social media utilization, recent research has started examining how bullying manifests virtually, cyberbullying. Notably, after the COVID‐19 pandemic, youth engagement on online platforms, one of the primary forms of social engagement, has increased (Zhang et al., 2021). The online world has also created novel mechanisms for bullying and victimization targeting minoritized groups, such as Latine LGBTQ+ youth in the United States (Abreu & Kenny, 2018; Giano et al., 2021). Unlike school‐based bullying, cyberbullying may extend beyond the school day and across settings, reinforcing identity‐based victimization in spaces where adolescents socialize, seek support, and explore their identities. However, research exploring cyberbullying experiences using an intersectional lens remains limited. Furthermore, cyberbullying may reflect distinct contextual features compared to school‐based bullying, including anonymity, persistence across settings, and broader audience exposure, which may differentially relate to internalized and emotional outcomes. Consistent with this distinction, differences in psychological impact from in‐person bullying versus cyberbullying for Latine LGBTQ+ youth in the United States have only begun to be explored (Rosales et al., 2023). These experiences also occurred within a broader U.S. sociopolitical context. During the last decade, many U.S. Latine LGBTQ+ adolescents were navigating heightened public debate surrounding LGBTQ+ rights in schools, increasing legislative efforts targeting LGBTQ+ youth, persistent anti‐immigrant rhetoric, and continued educational and social disruptions following the COVID‐19 pandemic. Together, these structural conditions likely shaped experiences of school climate, online engagement, perceived safety, belonging, and future opportunities.

### Beliefs about the future among Latine LGBTQ+ youth

For LGBTQ+ and Latine young people, bullying is rarely an isolated act; it often reflects broader systems of exclusion and discrimination that shape how they see themselves and what they believe is possible for their futures. During adolescence, individuals increasingly develop future‐oriented thinking, including expectations about educational, occupational, and relational trajectories. These expectations are shaped not only by individual psychological processes but also by social experiences and structural constraints (Hirsch et al., 2017). For Latine LGBTQ+ youth in the United States, these expectations develop within educational, community, and social contexts that may communicate both opportunities and barriers related to their intersecting identities. While research has long documented the emotional and psychological toll of bullying, far less is known about how these experiences are associated with youths' expectations, concerns, and sense of possibility regarding their future lives.

Bullying and other forms of marginalization can chip away at a young person's sense of agency and hope. Hirsch et al. (2017) found that LGBTQ+ individuals reported lower levels of hope and higher levels of hopelessness compared to their heterosexual and cisgender peers. These reductions in hope, defined as the capacity to set and pursue meaningful goals, help explain the elevated risk of depressive symptoms and suicidal behavior among LGBTQ+ individuals. The authors describe how persistent stigma and social rejection can “breed a sense of despair about the potential for the future to improve,” suggesting that oppression works not only through emotional pain but through disrupting how people imagine their futures. At the same time, expectations about the future may also reflect anticipated stigma and perceptions of structural barriers (e.g., discrimination in education or employment), rather than solely individual levels of hope or optimism. When multiple marginalized identities intersect, the impact may be compounded. Moe et al. (2025) found evidence that multiple forms of minority stress were associated with suicidal behavior in part through reduced hope. In other words, the cumulative weight of racism, heterosexism, and other *isms* can make it harder for youth to sustain optimism about the future or believe that their efforts will lead to meaningful change. This dynamic underscores how the psychological effects of bullying and discrimination are not only immediate but also long‐term, shaping how young people approach the future itself.

Facing social and political hostility, many LGBTQ+ youth in the United States enter adulthood with reduced optimism about personal, educational, and professional success. Yet, much of this research has centered on predominantly white samples, leaving significant gaps in understanding how these processes unfold among youth with intersecting marginalized identities. Latine LGBTQ+ youth in the United States, for instance, often navigate homophobia and transphobia within cultural contexts already shaped by racism, xenophobia, and socioeconomic inequality (Abreu et al., 2025, 2026; Barrita et al., 2026). Understanding these intersections is critical, as the loss of hope among Latine LGBTQ+ youth in the United States may reflect not just personal distress but the cumulative impact of structural inequities that signal to them that their futures are less valued. In the present study, “beliefs about the future” are conceptualized as a multidimensional construct capturing both (a) personal expectations about future opportunities (e.g., authenticity in work or education) and (b) perceptions of broader societal acceptance and anticipated discrimination. This framing reflects the combined influence of individual hope, anticipated stigma, and perceived structural barriers, rather than a singular measure of optimism.

### LGBTQ+ caregiver acceptance

It is critical to identify protective factors that can buffer the negative effects of bullying on the mental health of minoritized youth. Supportive social connections, particularly those characterized by acceptance and affirmation, have consistently demonstrated protective effects on the well‐being of marginalized youth facing bias‐based victimization (e.g., Espelage et al., 2019). Previous findings suggest that accepting caregiver relationships are associated with greater well‐being and may mitigate the effects of social stressors, including bullying, on mental health among LGBTQ+ youth (Needham & Austin, 2010). Among LGBTQ+ youth, caregiver acceptance has emerged as a particularly salient factor associated with lower levels of suicidality, depression, and anxiety, as well as greater self‐esteem (Needham & Austin, 2010; Ryan et al., 2010). These patterns are consistent with broader theoretical frameworks, including TIMSM, which emphasize the role of social support and affirming relationships as protective processes that can attenuate the impact of stressors over time (Rivas‐Koehl et al., 2023).

The evidence remains mixed regarding the role of caregiver acceptance as a buffer against bullying (Espelage et al., 2019). Abreu et al. (2026) found no moderating effect of caregiver acceptance on the association between bullying and well‐being among Latine LGBTQ+ youth in the United States. However, other studies (Abreu et al., 2025; Barrita, Abreu, et al., 2025; Barrita, Parmenter, et al., 2025) have found evidence of buffering effects from bullying experiences of Latine LGBTQ+ youth living in the United States. These inconsistencies suggest that the protective role of caregiver acceptance may vary depending on context (e.g., school vs. online environments), the type of outcome examined (e.g., internalized stigma vs. emotional distress), and the magnitude of the observed effects.

Latine LGBTQ+ youth in the United States, in particular, often experience compounded victimization related to both their sexual/gender identity and racial/ethnic identity (Zongrone et al., 2020), which can heighten risks for depressive symptoms and lower self‐esteem (Abreu et al., 2026; Toomey et al., 2018). Furthermore, among Latine LGBTQ+ youth, cultural values such as *familismo*, which emphasizes loyalty, interconnectedness, and closeness to family, have been highlighted (Abreu et al., 2022) as key to understanding how caregiver acceptance can serve as a protective factor against experiences of oppression, including school‐based bullying. However, it remains unclear whether this protective effect similarly extends to cyberbullying, a form of victimization that often occurs beyond the visibility of adults and caregivers. Given the pervasive nature of digital communication and the increased vulnerability of Latine LGBTQ+ youth in the United States to online harassment, understanding whether and how caregiver acceptance is associated with variation in the impact of cyberbullying is important to examine.

### The present study

**FIGURE 1:**
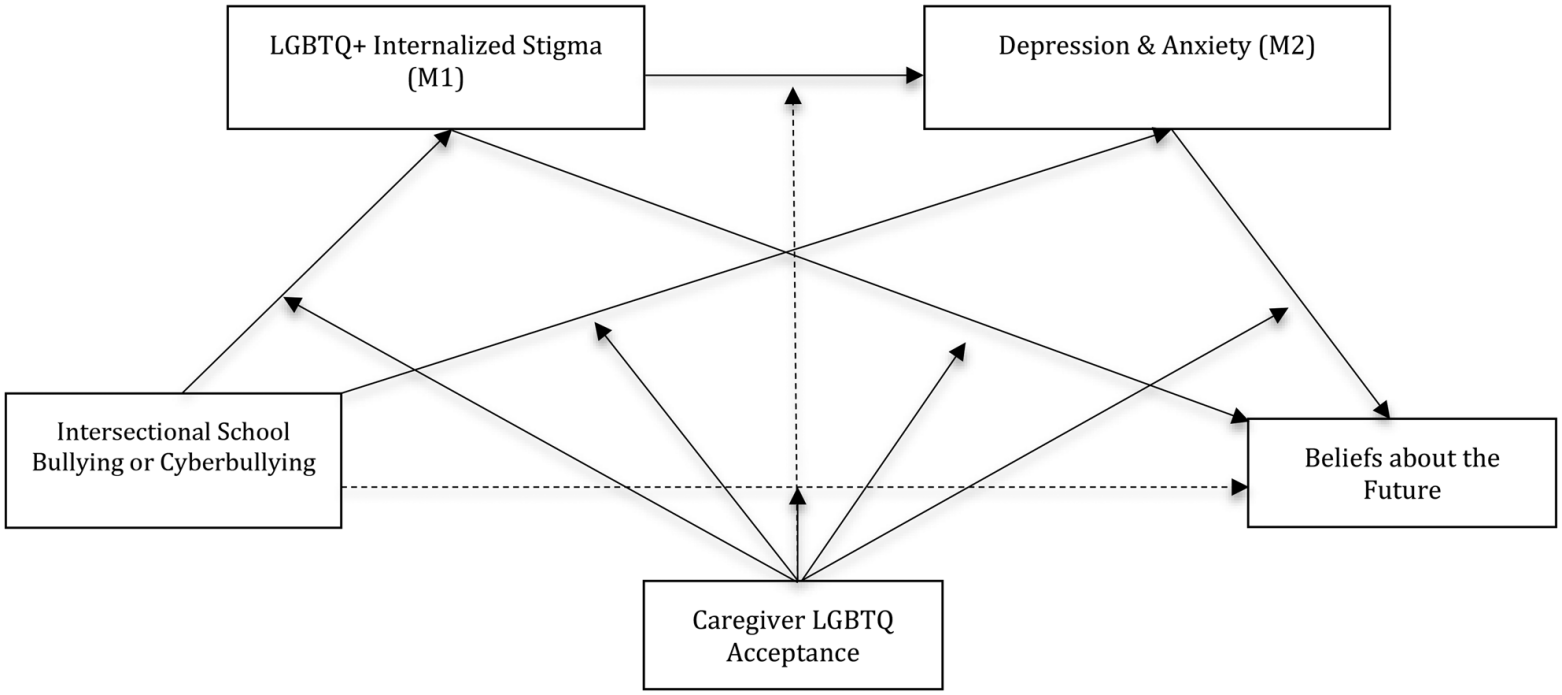
Conceptual model for the relation between Intersectional Bullying (School or Cyber) and Beliefs about the Future through LGBTQ+ Internalized Stigma and Depression & Anxiety, accounting for moderating effects from LGBTQ+ Caregiver Acceptance and controlling for Perceived Safety (School or Online) as covariate.

The current study examined associations among bullying experiences (both in school and online), LGBTQ+ internalized stigma, depression‐anxiety symptoms, and beliefs about the future as an LGBTQ+ person. In addition, we examined the potential buffering role of caregiver acceptance within these associations using a large national sample of Latine LGBTQ+ youth. Because these data were collected in 2022, the study should be understood within the broader U.S. sociopolitical and educational context described above. Consistent with an intersectional framework, bullying was operationalized as cumulative exposure to identity‐based victimization across multiple domains (e.g., race/ethnicity, sexual identity, gender, immigrant identity), reflecting the co‐occurrence of overlapping stressors rather than isolating single identity‐based experiences. Guided by MST and the TIMSM, our conceptual model (Figure 1) tested a set of theoretically informed associations. We hypothesized that intersectional bullying (school and cyber) would be associated with more negative beliefs about the future (H1a‐H1b), and with higher levels of LGBTQ+ internalized stigma (H2a‐H2b) and depression‐anxiety (H3a‐H3b). We further hypothesized that LGBTQ+ internalized stigma (H4a‐H4b) and depression‐anxiety (H5a‐H5b) would each be associated with more negative beliefs about the future. In addition, we tested whether patterns of association were consistent with a sequential indirect pathway linking intersectional bullying to beliefs about the future through LGBTQ+ internalized stigma and depression‐anxiety (H6a‐H6b).

In an exploratory analysis, we examined whether LGBTQ+ caregiver acceptance moderated associations across the model's direct and indirect pathways, given prior findings suggesting its potential protective role among Latine LGBTQ+ youth (Barrita, Parmenter, et al., 2025). Across models, perceived safety (school or online, depending on the context) was included as a covariate, as prior research suggests that perceptions of safety are associated with psychological distress in both school and digital environments (Barrita, Abreu, et al., 2025). Given the cross‐sectional nature of the data, the specified ordering of variables reflects a theoretically informed model rather than a test of temporal or causal processes. Accordingly, findings are interpreted as patterns of association consistent with, but not confirming, the proposed theoretical pathways.

## METHODS

### Participants and procedure

This online cross‐sectional study was conducted in accordance with ethical research principles and was approved by the University of Connecticut's IRB office as part of the larger national LGBTQ+ youth study collected in 2022 (Watson et al., 2024), which gathered information about LGBTQ+ youth systemic, institutional, and individual experiences and how these related to mental and physical health outcomes in the United States. Participants were 13–18 years of age, resided in the United States, and identified as a sexual and/or gender minority individual (i.e., did not identify as heterosexual and cisgender). Participants were presented with detailed study information as part of the informed consent process and were granted access to the survey only after providing consent. After completion, participants were offered a $5 Amazon or Starbucks gift card once their identity was verified via a valid school e‐mail or identification card check.

For the present analyses, we drew from the larger dataset to focus on participants who self‐identified as Latinx/a/o/e or Hispanic and reported being *out* (disclosing their LGBTQ+ identity) to at least one caregiver. This inclusion criterion was necessary for examining caregiver acceptance and should be considered when interpreting the scope of the findings. Data and materials for this study may be available from the corresponding author upon reasonable request. A total of 1795 participants met the inclusion criteria for this study. Of these, 35 were excluded for failing attention checks. The final sample included 1772 Latine LGBTQ+ youth (*M*_age_ = 15.82, *SD* = 1.49). Regarding racial identity, most participants identified as White (*n* = 767, 43.2%) or as “something else” (*n* = 543, 30.6%). In terms of gender identity, 351 participants (19.8%) identified as cisgender boys, 220 (12.4%) as cisgender girls, 344 (19.4%) as transgender boys, 112 (6.3%) as transgender girls, and 284 (16.0%) as nonbinary. Complete demographic information is presented in Table 1.

**TABLE 1:** Sociodemographic characteristics of participants.

Baseline characteristic	(*N* = 1772)	
	*n*	%
Racial identity		
White	767	43.2
Black or Afro	97	5.5
Native American	65	3.7
Asian	23	1.3
Multiracial	239	13.5
Something else	543	30.6
Did not disclose	38	2.2
Sexuality		
Gay/Lesbian	546	30.8
Bisexual	517	29.2
Queer	169	9.5
Pansexual	249	14.1
Asexual	128	7.2
Questioning	78	4.4
Something not listed	85	4.8
Gender identity		
Cis boy	351	19.8
Cis girl	220	12.4
Transgender boy	344	19.4
Transgender girl	112	6.3
Nonbinary	284	16.0
Genderfluid	149	8.4
Questioning	107	6.0
Gender non‐conforming	65	3.7
Genderqueer	64	3.6
Something else	70	4.0
Did not disclose	6	0.4

### Measures

#### Intersectional bullying (school and cyber)

To assess bullying, both in school and online, we adapted a previously validated weight‐related bullying measure (Puhl et al., 2012) by adding to each item the words “teased or mistreated by peers/classmates at school,” to capture bullying experiences in school, and the words “teased, harassed, or bullied online, virtually or electronically,” to capture cyberbullying experiences. Each version of the scale was presented separately in the survey. To apply an intersectional framework, both versions of the bullying measure (school or cyber) included eight items capturing multiple minoritized identities. Participants were asked, *“In the past 12 months, how often have you been teased or treated badly [wording adapted for in school or cyberbullying] because of your…”* followed by eight domains: weight, gender, race/ethnicity, sexual identity, religion, disability, immigrant identity, and masculinity/femininity. Responses were rated on a 5‐point Likert‐type scale ranging from 1 (*Never*) to 5 (*Very often*), with higher scores indicating greater exposure to bullying in the relevant context.

Scores were averaged across items to create composite indicators of intersectional school bullying and cyberbullying. These composite scores reflect the cumulative frequency of identity‐based victimization across domains, rather than isolating the effects of specific identity‐based experiences. This approach is consistent with prior work conceptualizing intersectional stress as the co‐occurrence of multiple forms of bias‐based victimization (Abreu et al., 2025; Barrita et al., 2026). At the same time, this operationalization may obscure variability across specific domains of victimization (e.g., race‐based vs. sexuality‐based bullying). As such, the composite scores should be interpreted as indicators of overall exposure to intersectional bullying rather than domain‐specific effects. Both versions of the scale demonstrated strong internal consistency in the present study (school‐bullying *α* = .84; cyberbullying *α* = .89) and have been previously validated with LGBTQ and BIPOC youth samples (Barrita et al., 2026).

#### LGBTQ+ internalized stigma

LGBTQ+ internalized stigma was assessed using a modified version of the *Internalized Homophobia Scale* (Shidlo, 1994). Item wording was adapted to reflect attitudes of acceptance, rejection, and pride related to both sexual and gender minority identities. Example items include, *“I wish I were not LGBTQ+.”* Participants rated their agreement with four items on a 4‐point Likert scale ranging from 1 (*Strongly disagree*) to 4 (*Strongly agree*), with higher scores indicating greater levels of LGBTQ+ internalized stigma. The measure demonstrated strong internal consistency in the current study (*α* = .82) and has been previously applied in research with BIPOC samples (Scanlon et al., 2024).

#### Depression and anxiety

Depression and anxiety were measured using the *Patient Health Questionnaire‐4* (PHQ‐4; Kroenke et al., 2009), which assesses the frequency of depressive and anxiety symptoms experienced over the past two weeks. The scale includes two items assessing depressive symptoms (*“Little interest or pleasure in doing things”* and *“Feeling down, depressed, or hopeless”*) and two items assessing anxiety symptoms (*“Feeling nervous, anxious, or on edge”* and *“Not being able to stop or control worrying”*). Participants rated each item on a 4‐point scale ranging from 0 (*Not at all*) to 3 (*Nearly every day*). Scores were averaged across all four items, with higher mean scores indicating greater depression and anxiety. The measure demonstrated strong internal consistency in the present study (*α* = .87) and has been widely validated across diverse populations (Kroenke et al., 2009).

#### Beliefs about the future as LGBTQ+

A novel measure developed as part of the larger study (Watson et al., 2024) was used to assess beliefs about the future as an LGBTQ+ individual. Five items were formulated to capture beliefs about the future at the societal level (“The LGBTQ community is accepted more every day by society”; “My LGBTQ identity will negatively affect my future” [reverse coded]) and the educational/career level (“I feel like I will be able to be my authentic self when applying for jobs in the future”; “My LGBTQ identity will negatively affect my future college and higher education opportunities” [reverse coded]; “I fear I will be discriminated against at work in the future because I'm LGBTQ” [reverse coded]). Each item was rated on a 5‐point Likert scale from 1 = strongly disagree to 5 = strongly agree. Mean scores were calculated, with higher scores indicating more positive beliefs about the future as an LGBTQ+ individual. The measure demonstrated strong internal consistency in the present study (α = .85). Conceptually, this measure reflects a multidimensional, future‐oriented construct that encompasses personal expectations regarding authenticity and opportunity, perceptions of broader societal acceptance, and anticipated discrimination. Accordingly, it should be interpreted as capturing beliefs about the future in relation to one's LGBTQ+ identity rather than as a singular measure of hope or optimism.

#### LGBTQ+ caregiver acceptance

Caregivers' acceptance and rejection of LGBTQ+ youth were measured using three items from the *LGBTQ+ Parental Acceptance/Rejection Scale* (Pollitt et al., 2023) that assessed accepting behaviors. This scale evaluates the frequency and degree of acceptance or rejection expressed by parents or caregivers regarding a participant's LGBTQ+ identity. Example items include, *“My parents say that they like me as I am in regard to being an LGBTQ+ person,”* and *“My parents say they are proud of me for being an LGBTQ+ person.”* Participants rated each item on a 4‐point Likert scale ranging from 1 (*Never*) to 4 (*Often*), with higher scores indicating greater caregiver acceptance. The measure demonstrated excellent internal consistency in the present study (*α* = .93) and has been previously validated with Latine LGBTQ+ youth (Barrita, Abreu, et al., 2025).

#### Perceived safety

##### School safety

Perceived school safety was measured using a series of nine items that asked: “*While at school, how often do you feel safe*…” followed by various settings: “*in classroom, in the bathroom, in the locker room, in the hallways, in the library, in the cafeteria, outside of school grounds, getting to/from school, on the school bus*.” Each item was rated using a 4‐point Likert scale (0 = *Not safe at all*, 3 = *Very safe*), and mean scores were calculated, with higher scores representing greater perceived school safety. The measure demonstrated excellent internal consistency in the present study (*α* = .90).

##### Online safety

Perceived online safety was measured using the question, *“How safe do you feel when you participate in online activities (e.g., gaming, social media, texting, chatting)?”* Responses were recorded on a 4‐point Likert scale (0 = *Not safe at all*, 3 = *Very safe*), with higher scores representing greater perceived online safety.

#### Demographics

We used a combination of open‐ended and multicategory questions to assess participants' demographics, including gender, sexual and racial/ethnic identity, and age. For Latine/x/o/a or Hispanic identity, we asked participants to respond yes or no to the question “Do you identify as Hispanic, Latino/a/x/e?”

### Analyses

All analyses were conducted using SPSS Version 28.0. Preliminary analyses were performed to examine data distributions and assess statistical assumptions. To test the two hypothesized models, we used PROCESS (Hayes, 2012) and conducted a serial indirect‐effects analysis (Model 6) with bootstrapping (10,000 samples). Both models differed only by the predictor, intersectional bullying (school bullying vs. cyberbullying). Consistent with the study's cross‐sectional design, these models were specified as tests of theoretically informed indirect associations rather than causal or temporal processes. Finally, in an exploratory approach, we also tested whether LGBTQ+ caregiver acceptance moderated the indirect effects within this serial indirect‐effects model across all paths using PROCESS Model 92 (Hayes, 2012), which tests significant interactions across all indirect and direct paths. All models included perceived safety (school or online, accordingly) as a covariate. Importantly, we note that the proposed sequence was theory‐driven and intended to evaluate whether the observed associations were consistent with MST‐ and TIMSM‐informed pathways.

### Positionality statement

Our research team brings intersecting personal and professional identities that inform our approach to this work. The first three authors identify as Latine Immigrants and Queer early‐career psychologists. Our personal and professional experiences navigating intersecting systems of marginalization inform our commitment to conducting research that uplifts and represents the complexities of Latine LGBTQ+ lives. The fifth author identifies as a first‐generation Latine graduate student. These positionalities ground our interpretations in an awareness of cultural resilience, systemic inequities, and the importance of community‐based perspectives. The fourth and sixth authors identify as Queer white psychologists, one early career and one mid‐career, whose research and practice focus on the well‐being of LGBTQ+ minority populations. Their positionality offers both insider perspectives on Queer identities and reflexive awareness of the privileges associated with whiteness within academic and clinical spaces. Further, the entire authorship team has experience in intersectional Black Feminist scholarship that critiques systems of injustice while advocating for transformative change that empowers Latine LGBTQ+ people. These positionalities also informed our analytic decisions, including the use of intersectional frameworks, attention to cumulative forms of oppression, and cautious interpretation of findings within broader structural and developmental contexts. They further shaped our commitment to avoiding deficit‐only narratives by considering resilience, protective factors, and community‐relevant implications for Latine LGBTQ+ youth. Together, we share a commitment to advancing equity, representation, and social justice in psychological research and recognize how our positionalities influence the questions and the interpretations we make in this study.

## RESULTS

A preliminary analysis was conducted to verify the normality of our main variables, identify missing data, and detect significant outliers. A small proportion of participants (1.2%) had missing data on the main study variables. Because the proportion of missingness was below 5% and appeared to be missing at random, we addressed it using multiple imputation procedures. Assumptions for the main analyses (e.g., homoscedasticity, multicollinearity, and independence of errors) were examined, and no significant concerns were identified. Potential covariates were evaluated prior to the primary analyses by examining bivariate associations (for continuous variables) and group differences (for categorical variables) using ANOVAs, with both the predictor and outcome variables. Consistent with recommended covariate selection practices favoring theoretically relevant and empirically associated variables (Miller & Chapman, 2001), only perceived safety (school or online, depending on the model) demonstrated consistent associations and was therefore retained as a covariate in the final analyses.

Intersectional school bullying and intersectional cyberbullying were moderately correlated in the present sample (*r* = .659, *p* < .001), suggesting related but empirically distinct forms of victimization. As part of the review process, we conducted post hoc descriptive analyses examining correlations between individual bullying items and key study outcomes. These results are presented in Table S1 to provide additional context on how specific forms of bullying may relate differentially to psychosocial functioning.

### Serial indirect effects of intersectional bullying

Two independent serial mediation models (PROCESS Model 6; Hayes, 2012) were conducted to examine the indirect and total associations of intersectional bullying (school‐based and cyber) on beliefs about the future, through LGBTQ+ internalized stigma and depression‐anxiety, while controlling for perceived safety (school or online, accordingly) as the only covariate. All coefficients are standardized.

#### Model 1: Intersectional school bullying

Controlling for perceived school safety, a significant indirect effect was found from intersectional school bullying to LGBTQ+ internalized stigma, *B* = 0.12, SE = 0.02, *p* < .001, 95% CI [0.07, 0.17], indicating that greater intersectional school bullying was associated with higher levels of LGBTQ+ internalized stigma (H2a). LGBTQ+ internalized stigma was, in turn, negatively associated with beliefs about the future (H4a), *B* = −0.26, SE = 0.01, *p* < .001, 95% CI [−0.30, −0.22]. Intersectional school bullying was also associated with higher depression‐anxiety (H3a), *B* = 0.22, SE = 0.02, *p* < .001, 95% CI [0.08, 0.31], and depression‐anxiety was negatively associated with beliefs about the future (H5a), *B* = −0.06, SE = 0.01, *p* < .001, 95% CI [−0.09, −0.03]. LGBTQ+ internalized stigma also showed a significant positive association with depression‐anxiety, *B* = 0.12, SE = 0.03, *p* < .001, 95% CI [0.09, 0.21]. These results are consistent with an indirect pathway in which higher intersectional school bullying is associated with negative beliefs about the future through higher LGBTQ+ internalized stigma and depression‐anxiety.

The direct effect of intersectional school bullying on beliefs about the future was not statistically significant (H1a), *c* = −0.03, SE = 0.02, *p* = .179, 95% CI [−0.06, 0.01]. However, the total effect was significant and negative, *c'* = −0.07, SE = 0.02, *p* < .001, 95% CI [−0.11, −0.03], indicating that intersectional school bullying was associated with negative beliefs about the future when accounting for indirect associations through LGBTQ+ internalized stigma and depression‐anxiety (H6a).

#### Model 2: Intersectional cyberbullying

A similar pattern of findings emerged in the intersectional cyberbullying model. Controlling for perceived online safety, intersectional cyberbullying was positively associated with LGBTQ+ internalized stigma (H2b), *B* = 0.10, SE = 0.02, *p* < .001, 95% CI [0.06, 0.13], which was again negatively associated with beliefs about the future (H4b), *B* = −0.31, SE = 0.02, *p* < .001, 95% CI [−0.35, −0.27]. Intersectional cyberbullying also showed a positive association with depression‐anxiety (H3b), *B* = 0.12, SE = 0.03, *p* < .001, 95% CI [0.07, 0.18], while depression‐anxiety was negatively associated with beliefs about the future (H5b), *B* = −0.08, SE = 0.02, *p* < .001, 95% CI [−0.11, −0.04]. LGBTQ+ internalized stigma was also significantly related to depression‐anxiety, *B* = 0.26, SE = 0.03, *p* < .001, 95% CI [0.20, 0.32], providing further evidence for the indirect pathway.

The direct effect of intersectional cyberbullying on beliefs about the future was not significant (H1b), *c* = −0.02, SE = 0.02, *p* = .221, 95% CI [−0.05, 0.01]. However, the total effect was significant and negative, *c'* = −0.06, SE = 0.02, *p* < .001, 95% CI [−0.09, −0.03], indicating that greater intersectional cyberbullying was associated with negative beliefs about the future when accounting for indirect associations through LGBTQ+ internalized stigma and depression‐anxiety (H6b).

### Interactions with LGBTQ+ caregiver acceptance

In an exploratory approach, two moderated serial mediation models, using 10,000 bootstrap samples and the PROCESS macro (Model 92; Hayes, 2012), were conducted to test the buffering effects of LGBTQ+ caregiver acceptance in our serial indirect‐effect models. Only statistically significant effects are reported below; all coefficients are presented in their standardized form (see Table 2 for full results).

**TABLE 2:** Moderated mediation model characteristics for predictors on internalized LGBT stigma (mediator 1), depression (mediator 2), and beliefs about the future (dependent variable).

Model 1	Internalized LGBTQ stigma			Depression & Anxiety			Beliefs about the future		
School	B	SE	95% CI	B	SE	95% CI	B	SE	95% CI
School bullying	0.007	0.053	−0.111; 0.097	**0.382** ***	0.066	0.253; 0.512	−0.051	0.045	−0.140; 0.038
Int. stigma (M1)				**0.199** **	0.064	0.073; 0.325	**−0.228** ***	0.042	−0.311; −0.145
D‐A (M2)							−0.063	0.035	−0.131; 0.005
Caregiver A. (W)	−0.006	0.046	−0.096; 0.085	**−0.289** ***	0.078	−0.441; −0.136	0.005	0.053	−0.109; 0.098
X × W	**−0.050** *	0.012	−0.095; −0.005	**−0.064** *	0.029	−0.121; −0.008	0.014	0.020	−0.025; 0.053
M1 × W				−0.033	0.032	−0.094; 0.029	−0.017	0.021	−0.058; 0.024
M2 × W							0.004	0.018	−0.031; 0.039
Model R^2^	**0.091, *F*(4,1691) = 42.22, *p* < .0001**			**0.194, *F*(6,1689) = 67.65, *p* < .001**			**0.211, *F*(8,1687) = 56.48, *p* < .0001**		
Interaction Δ^2^	**0.003, *F*(1,1691) = 4.61, *p* < .05**			**0.003, *F*(1,1689) = 6.64, *p* < .05**			0.001, *F*(1,71,687) = 0.57, *p* = .48		

Model 2	Internalized LGBTQ stigma			Depression			Feelings about the future		
Cyber	B	SE	95% CI	B	SE	95% CI	B	SE	95% CI
Cyberbullying	**0.065** **	0.009	0.012; 0.144	**0.425** ***	0.060	0.308; 0.542	**−0.098** *	0.004	−0.176; −0.019
Int. stigma (M1)				**0.222** ***	0.063	0.098; 0.346	**−0.288** ***	0.042	−0.370; −0.207
Depression (M2)							**−0.096** **	0.033	−0.161; −0.031
Caregiver A. (W)	0.037	0.041	−0.044; 0.118	**−0.312** ***	0.075	−0.458; −0.166	0.052	0.052	−0.153; 0.049
X × W	**−0.046**	0.002	−0.098; −0.004	**−0.086** **	0.028	−0.140; −0.032	0.010	0.018	−0.026; 0.046
M1 × W				−0.011	0.031	−0.072; 0.051	0.004	0.021	−0.045; 0.036
M2 × W							0.012	0.017	−0.021; 0.046
Model R^2^	**0.063, *F*(4,1691) = 41.56, *p* < .0001**			**0.135, *F*(6,1689) = 58.02, *p* < .001**			**0.186, *F*(8,1687) = 60.72, *p* < .0001**		
Interaction Δ^2^	**0.003, *F*(1,1691) = 4.51, *p* <**. **05**			**0.015, *F*(1,1689) = 9.67, *p* < .01**			0.001, *F*(1,1687) = 0.51, *p* = .58		

*Note*: Bold values indicates statistically signifcant.

Abbreviations: Caregiver A., caregiver LGBTQ acceptance; D‐A < Depression & Anxiety; Int. stigma, internalized LGBTQ stigma; M, mediator 1 or 2; W, moderator.

* *p* < .05.

** *p* < .01.

*** *p* < .001.

#### Model 1: Intersectional school bullying

In the intersectional school‐bullying model, LGBTQ+ caregiver acceptance significantly moderated the association between intersectional school bullying and LGBTQ+ internalized stigma, *B* = −0.050, SE = 0.012, 95% CI [−0.095, −0.005], such that higher caregiver acceptance was associated with a weaker positive association between bullying and internalized stigma. LGBTQ+ caregiver acceptance also moderated the path from intersectional school bullying to depression‐anxiety, *B* = −0.064, SE = 0.029, 95% CI [−0.121, −0.008], suggesting a buffering effect of LGBTQ+ caregiver acceptance on the mental health outcomes from school‐based victimization. Consistent with the original model, LGBTQ+ internalized stigma remained significantly associated with increased depression‐anxiety (*B* = 0.199, SE = 0.064, 95% CI [0.073, 0.325]) and beliefs about the future (*B* = −0.228, SE = 0.042, 95% CI [−0.311, −0.145]). The direct association between intersectional school bullying and beliefs about the future was not statistically significant in the moderated model (*B* = −0.051, *SE* = 0.045, 95% CI [−0.140, 0.038]), consistent with the non‐significant direct association observed in the original model.

#### Model 2: Intersectional cyberbullying

In the intersectional cyberbullying model, LGBTQ+ caregiver acceptance significantly moderated the path from intersectional cyberbullying to depression‐anxiety, *B* = −0.086, SE = 0.028, 95% CI [−0.140, −0.032), such that higher caregiver acceptance was associated with a weaker positive association between cyberbullying and depression‐anxiety. The interaction between intersectional cyberbullying and caregiver acceptance in predicting LGBTQ+ internalized stigma was statistically significant (*B* = −0.046, *SE* = 0.002, 95% CI [−0.098, −0.004]), although the confidence interval suggests a small effect size and should be interpreted with caution. Consistent with the original model, LGBTQ+ internalized stigma remained positively associated with depression‐anxiety (*B* = 0.222, *SE* = 0.063, 95% CI [0.098, 0.346]), and both LGBTQ+ internalized stigma (*B* = −0.288, *SE* = 0.042, 95% CI [−0.370, −0.207]) and depression‐anxiety (*B* = −0.096, *SE* = 0.033, 95% CI [−0.161, −0.031]) were associated with more negative beliefs about the future. The direct association between intersectional cyberbullying and beliefs about the future remained statistically significant in the moderated model (*B* = −0.098, *SE* = 0.004, 95% CI [−0.176, −0.019]).

**FIGURE 2:**
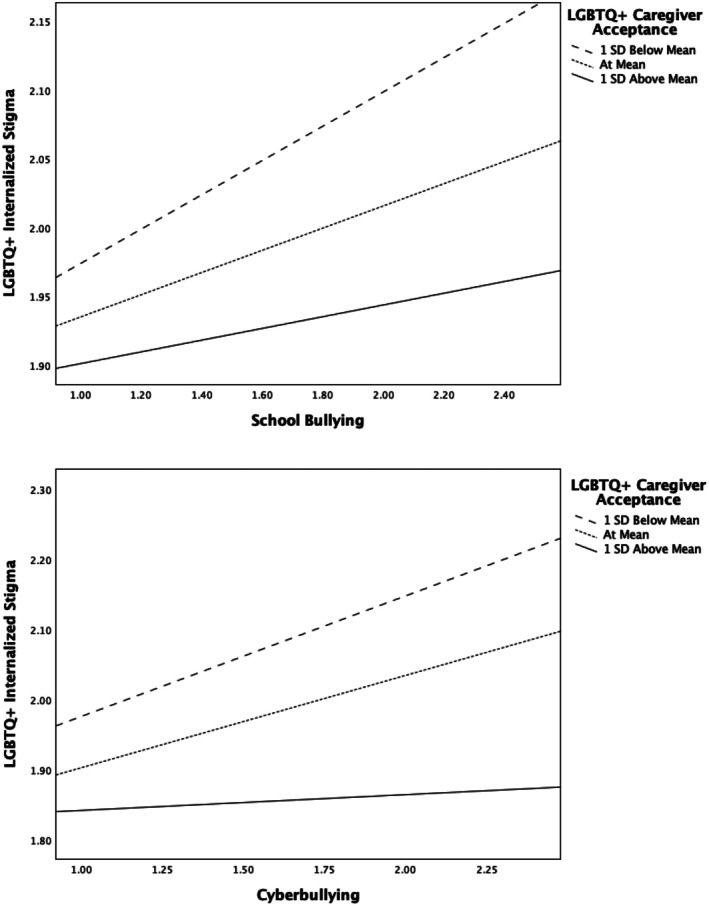
Moderating influence of LGBTQ+ Caregiver Acceptance on the association of Intersectional Bullying (School or Cyber) and LGBTQ+ Internalized Stigma.

**FIGURE 3:**
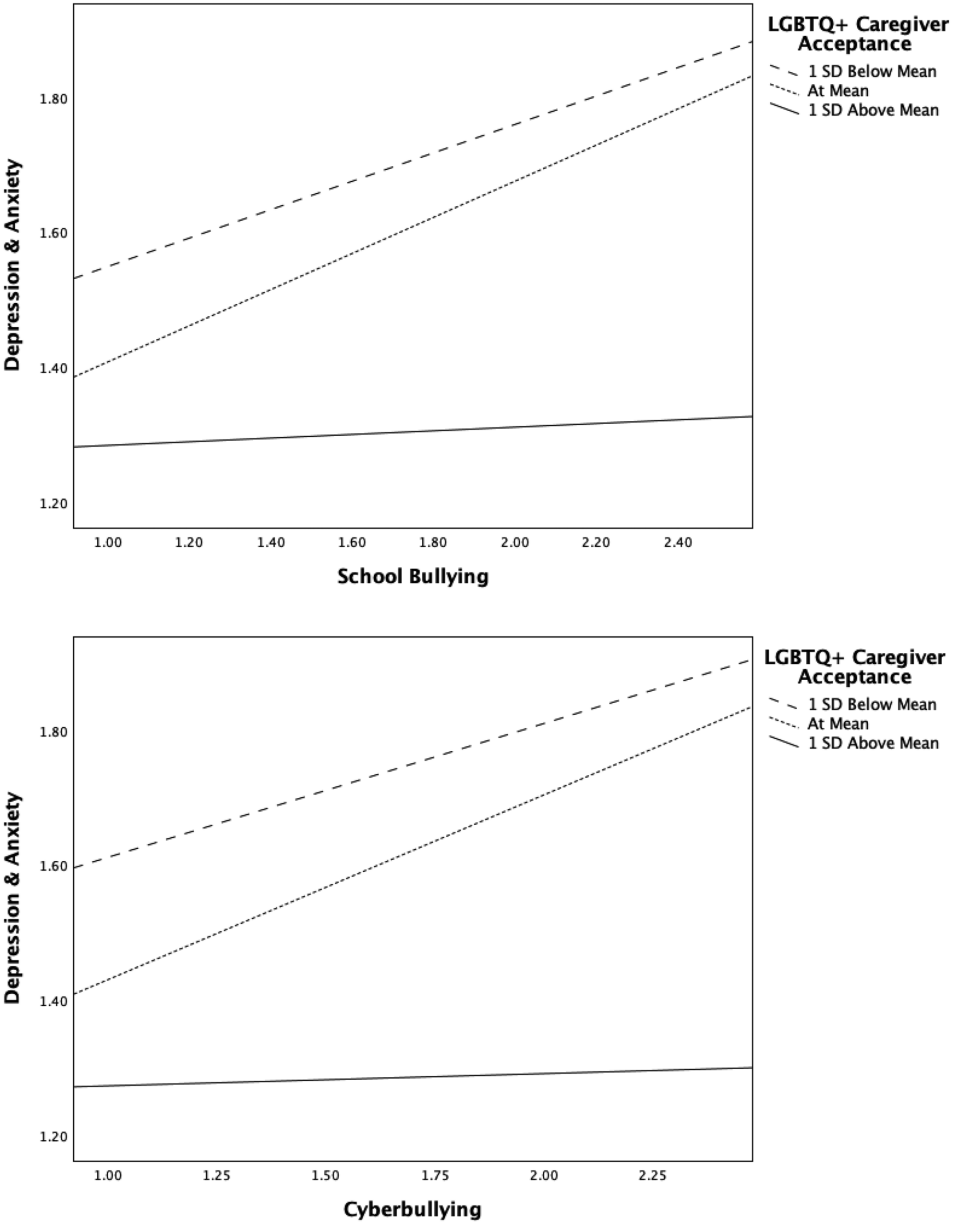
Moderating influence of LGBTQ+ Caregiver Acceptance on the association of Intersectional Bullying (School or Cyber) and Depression & Anxiety.

For the intersectional school‐bullying model (Figure 2), the association between school bullying and LGBTQ+ internalized stigma was strongest at low levels of caregiver acceptance and progressively weaker at mean and high levels. Similarly, the association between school bullying and depression‐anxiety was attenuated at higher levels of caregiver acceptance. Although these patterns are consistent with a buffering interpretation, the differences in slopes were modest in magnitude, indicating that caregiver acceptance reduced, but did not eliminate, the associations between bullying and psychological outcomes. For the intersectional cyberbullying model (Figure 3), a similar pattern emerged for the association between cyberbullying and depression‐anxiety, such that the slope was steeper at lower levels of caregiver acceptance and weaker at higher levels. However, the magnitude of this interaction effect was relatively small, and the overall pattern of associations remained consistent across levels of caregiver acceptance. Notably, the interaction between cyberbullying and caregiver acceptance predicting LGBTQ+ internalized stigma, although statistically significant, was characterized by a very small standard error and effect size, warranting cautious interpretation. Across both models, these simple slope analyses suggest that caregiver acceptance is associated with modest attenuation of the associations between intersectional bullying and psychological distress. However, the practical significance of these interaction effects appears limited, as differences in slopes across levels of caregiver acceptance were relatively small and did not substantially alter the overall pattern of associations.

## DISCUSSION

The present study offers compelling evidence that intersectional school‐based and cyberbullying were associated with U.S. Latine LGBTQ+ youths' beliefs about their future, primarily through the internalization of LGBTQ+ stigma and heightened negative psychological symptoms. Although the direct effects from either form of intersectional bullying on beliefs about the future as an LGBTQ+ person were non‐significant, the robust indirect pathways underscore the critical psychological mechanisms, particularly LGBTQ+ internalized stigma, that may help explain how external victimization is associated with diminished hope and optimism. Given the cross‐sectional design, these findings should not be interpreted as causal or temporally fixed. It is also plausible that youth experiencing greater depressive symptoms may perceive victimization more frequently or hold more negative future beliefs, suggesting potentially reciprocal processes that warrant longitudinal investigation. This aligns with prior research demonstrating that exposure to identity‐based bullying contributes to reduced well‐being among LGBTQ+ youth (Abreu et al., 2026; Gower et al., 2018; Toomey et al., 2018), with even more pronounced effects among Latine LGBTQ+ youth in the United States (Barrita, Parmenter, et al., 2025; Rosales et al., 2023), highlighting how exposure to bias‐based bullying may be linked to internalized shame and emotional distress among Latine LGBTQ+ youth. Within the U.S. context, where school and online environments are important developmental settings for adolescents, these findings suggest that repeated exposure to intersectional bullying may shape not only current psychological well‐being but also how youth envision future educational, occupational, and social opportunities.

Nevertheless, meaningful differences emerged between intersectional school‐based and cyberbullying models. Intersectional school bullying appeared to be more strongly associated with depression‐anxiety symptoms, perhaps reflecting the relational proximity and repeated exposure that occur in face‐to‐face peer environments, even after accounting for perceived school safety. For many Latine LGBTQ+ youth in the United States, schools are central developmental settings where peer relationships, identity development, and access to affirming resources intersect. Consequently, experiences of intersectional bullying within these environments may be particularly salient because they occur in settings intended to support adolescents' learning, development, and well‐being. School‐based victimization may therefore reflect not only interpersonal peer aggression but also broader school environments in which experiences of safety and belonging differ across students (Abreu et al., 2026). These daily, observable interactions may heighten emotional exhaustion and erode beliefs of safety and belonging.

By contrast, intersectional cyberbullying showed somewhat stronger associations with LGBTQ+ internalized stigma. The constant, boundless nature of online victimization, in which harassment can follow youth beyond school walls and persist in digital spaces, may amplify internalization processes (Abreu & Kenny, 2018). Online environments also allow for anonymity and a wider audience, factors that can intensify the humiliation and perceived uncontrollability of victimization. For U.S. adolescents, digital environments represent an increasingly important context for identity exploration and peer connection. Consequently, identity‐based harassment occurring online may reinforce negative self‐perceptions even after youth leave school settings. From the perspective of the MST framework (Meyer, 2003), these digital interactions serve as powerful distal stressors that continually reinforce stigma messages and maintain proximal stress through ongoing self‐surveillance and fear of exposure. Thus, while both intersectional bullying contexts activated similar stress mechanisms, their qualitative contours differed, given that intersectional school bullying seemed to erode emotional well‐being through interpersonal rejection, whereas cyberbullying more deeply shaped identity processes through chronic, internalized stigma.

These findings underscore the importance of considering the unique ecological environments in which different forms of intersectional bullying unfold. Because these data were collected in 2022, the findings should be interpreted within the broader U.S. sociopolitical and educational context described earlier. They should also be understood within adolescence as a developmental period marked by rapid identity development, shifting peer salience, and increasing future orientation. Accordingly, internalized stigma, emotional distress, and beliefs about the future may be especially dynamic between early and late adolescence. School‐based victimization, though damaging, occurs within systems where interventions, peer education, and teacher training can directly target bias and promote inclusive climates. By contrast, intersectional cyberbullying represents a diffuse, often invisible form of intersectional harm that requires broader digital literacy and community‐based strategies to counteract. Both forms of intersectional bullying, however, converge in their cumulative toll on Latine LGBTQ+ youths' psychological well‐being and perceived life chances, echoing previous research showing that chronic exposure to discrimination erodes hope and heightens despair among multiply marginalized youth (Hirsch et al., 2017; Moe et al., 2025).

Interpreting these findings through MST and TIMSM (Meyer, 2003; Rivas‐Koehl et al., 2023) reveals the compounding effects of multiple, time‐extended stressors. MST emphasizes how distal experiences, such as bullying, are internalized, while TIMSM further elucidates how these processes are cumulative and context‐dependent over time and across social settings. In our data, the persistence of stigma across both in‐person and virtual spaces is consistent with TIMSM's core principle: That intersectional stress is not episodic but recursive, re‐emerging in new forms across contexts. For Latine LGBTQ+ youth in the United States, experiences of racism, xenophobia, and cisheterosexism may compound across school and online domains, deepening the psychological imprint of marginalization and extending its influence into future expectations and self‐concept. These findings are particularly relevant within U.S. schools and digital environments, where adolescents navigate identity development while encountering varying levels of affirmation, safety, and support. Although temporal unfolding and recursive processes could not be directly tested in the present study, the observed patterns align with TIMSM's proposition that minority stress processes operate across contexts and may accumulate over time.

The inclusion of LGBTQ+ caregiver acceptance further illuminated a protective dimension within these stressors. Consistent with prior work documenting the mental health benefits of caregiver affirmation among LGBTQ+ youth (Ryan et al., 2010), our results revealed that caregiver acceptance buffered the effects of bullying on both internalized stigma and depression‐anxiety. These findings suggest that affirming family environments can disrupt the internalization of societal bias, thereby preventing external oppression from translating into self‐stigma and emotional distress. However, these interaction effects were modest in magnitude, indicating attenuation rather than elimination of risk. Thus, caregiver acceptance should be interpreted as a meaningful but not sufficient protective factor within a broader ecological network of supports for Latine LGBTQ+ youth in the United States. This moderating effect is especially meaningful in the context of Latine cultural values such as familismo (Abreu et al., 2022). For many Latine LGBTQ+ youth, acceptance from caregivers not only validates their identity but also reinforces a sense of belonging within a cultural framework that might otherwise conflict with LGBTQ+ identities. Our findings thus build upon prior mixed evidence regarding caregiver support (Espelage et al., 2019), suggesting that while the protective effects of acceptance may vary across contexts, they play a particularly crucial role for Latine LGBTQ+ youth in the United States experiencing intersectional bullying. These results also align with TIMSM's emphasis on protective temporal buffers, such as family affirmation, that can interrupt the cumulative stress trajectories associated with intersecting oppressions. Although statistically significant, differences across simple slopes were relatively small, suggesting that the practical significance of these buffering effects may be incremental rather than transformative. Even so, modest protective effects may remain meaningful when experienced repeatedly across adolescence.

Taken together, our findings show how intersectional bullying is associated with psychological functioning and beliefs about the future among Latine LGBTQ+ youth in the United States. The results highlight that the harm of intersectional bullying is not confined to immediate emotional distress but extends to shaping how youth imagine their long‐term possibilities, a process shaped by the interplay between external bullying, internalized stigma, and mental health. This is consistent with prior research showing that persistent discrimination and stigma reduce hope and optimism, often leading to a “sense of despair about the potential for the future to improve” among LGBTQ+ individuals (Hirsch et al., 2017; Moe et al., 2025). For Latine LGBTQ+ youth in the United States, this loss of hope may be particularly acute due to the simultaneous experience of racial/ethnic marginalization, heterosexism, and the structural inequities that signal limited opportunity (Barrita, Abreu, et al., 2025). Because these findings reflect the experiences of adolescents living in the United States, they should be interpreted within the educational, social, and cultural contexts in which these youth navigate intersecting forms of marginalization. At the same time, U.S. Latine LGBTQ+ youth are not a monolithic population. Experiences likely vary across racial identity, immigration history, language context, gender identity, geography, and access to affirming resources. Future research using larger and more evenly distributed subgroup‐specific samples is needed to examine whether the frequency, forms, and psychological correlates of bullying differ across demographic subgroups within Latine LGBTQ+ youth populations.

### Clinical implications

Clinicians working with Latine LGBTQ+ youth in the United States should attend to the intersectional nature of stress exposure. These recommendations are informed by the observed associations in the present study and broader prior literature, rather than direct tests of intervention efficacy. Therapeutic interventions must explicitly address how racism, xenophobia, and cisheterosexism co‐occur and manifest through both in‐person and online experiences. Cognitive‐behavioral and narrative approaches can be adapted to help youth identify and challenge internalized stigma, foster self‐compassion, and reconstruct future‐oriented hope that intersectional bullying may have eroded (Burger & Pachankis, 2024). For clinicians practicing in the United States, these approaches may be especially important given that many Latine LGBTQ+ youth navigate intersecting stressors across school, family, community, and digital environments. Incorporating family members, particularly caregivers, into treatment can further enhance outcomes. Family therapy models that integrate cultural values such as familismo can help caregivers understand the significance of affirmation, while simultaneously addressing potential conflicts between traditional beliefs and LGBTQ+ acceptance. Clinicians should also incorporate digital mental health literacy into their practice. Given the heightened role of intersectional cyberbullying, mental health providers can support clients in developing strategies for online boundary‐setting, reporting mechanisms, and critical engagement with social media. Group therapy or peer support programs can also create affirming spaces for Latine LGBTQ+ youth in the United States to process experiences of digital victimization collectively, reducing isolation and reinforcing shared resilience.

### School‐settings implications

Schools remain central settings for prevention and early intervention. Although the present study did not directly assess school climate or institutional policies, the associations involving school‐based bullying support the relevance of school settings as important intervention contexts. Educators and administrators must move beyond generic anti‐bullying policies to adopt intersectional, culturally responsive frameworks that explicitly recognize and address the compounded discrimination faced by Latine LGBTQ+ students. Professional development for teachers and counselors should emphasize the identification of intersectional bullying behaviors, including microaggressions and racialized forms of exclusion. Because students' perceptions of safety and belonging are shaped by both peer interactions and the broader school environment, these efforts should also promote identity‐affirming practices that foster inclusive school climates. Integrating LGBTQ+ and Latine histories and narratives into curricula can further cultivate affirmation and belonging. School‐based mental health professionals can use these findings to advocate for trauma‐informed, identity‐affirming support systems. On‐campus counseling centers should be trained to recognize the dual burdens of cultural and sexual/gender‐based stigma and provide multilingual, culturally congruent services for students and families. Partnerships with community organizations serving Latine and LGBTQ+ populations can extend support beyond the school walls, ensuring continuity of care. Although the clinical and school‐setting implications we name are most directly applicable to Latine LGBTQ+ youth in the United States, they underscore the broader importance of culturally responsive, identity‐affirming approaches that recognize and address intersecting forms of identity‐based victimization.

### Limitations and future studies

Our findings must be interpreted in consideration of several limitations. First, none of our findings can be interpreted as causal, as our sample was collected at a single point in time. Additionally, our serial indirect‐effect and moderated mediation models, which are specified with a specific ordering of variables, assume a theoretical and conceptual linear relation in which intersectional bullying occurred before the other outcome variables. However, these directions were not explicitly tested in our methods. Nonetheless, recommendations for indirect‐effect analysis using cross‐sectional data support this analytical approach, particularly for hard‐to‐reach populations and phenomena that may not be feasible to capture with longitudinal or experimental designs (Hayes & Rockwood, 2020). Previous research (Abreu et al., 2025; Barrita, Parmenter, et al., 2025) conducted by most of the authors in this study has tested similar mediation and moderation models among LGBTQ+ youth using cross‐sectional data.

Future studies should explore these associations using longitudinal approaches to assess temporal ordering and potential causal effects. Future longitudinal research should also test alternative and reciprocal pathways, including whether depression‐anxiety shapes perceptions of bullying or future beliefs over time. Similarly, future research using larger and more evenly distributed subgroup‐specific samples would be well positioned to examine whether the frequency, forms, and psychological correlates of bullying vary across racial/ethnic, gender, and other demographic subgroups within U.S. Latine LGBTQ+ youth populations. Our measures were adapted to capture bullying experiences, both in‐person and virtual, based on multiple minoritized identities. However, these adaptations do not capture every type of minoritized identity; therefore, they are limited in their ability to account for the full range of experiences of identity‐based bullying within the sample. Future studies focusing on nuance in intersectional bullying, protective factors, and psychological outcomes should adapt their methods to capture the complex aspects of the overall experience and the humanity of minoritized communities (Garcini et al., 2025). Additionally, participants were recruited online, required internet access, and had to be out to at least one caregiver to be included in analyses involving caregiver acceptance. As such, the sample may underrepresent youth who are more socially isolated, less supported at home, unstably housed, or not yet disclosed to caregivers. These sampling characteristics constrain the generality of the findings. The results are most directly applicable to Latine LGBTQ+ adolescents aged 13–18 who were living in the United States in 2022, had internet access, and had disclosed their LGBTQ+ identity to at least one caregiver. They may be less applicable to U.S. Latine LGBTQ+ youth who have not disclosed their identity, lack stable internet access or housing, or experience limited caregiver support. The findings should not be generalized to LGBTQ+ youth living outside the United States, including youth in Latin America or other global contexts, where legal protections, educational systems, immigration experiences, cultural norms, and sociopolitical conditions differ substantially. Finally, because the beliefs about the future measure is novel and multidimensional, future research should further evaluate its dimensionality and construct validity across diverse LGBTQ+ youth populations within the United States and beyond.

## CONCLUSION

In sum, this study highlights that both intersectional school‐based and cyberbullying experiences are meaningfully associated with the mental health and future beliefs of Latine LGBTQ+ youth in the United States, though through distinct pathways. Intersectional school bullying was more closely associated with emotional well‐being, while intersectional cyberbullying was more strongly associated with internalized stigma. Across both contexts, caregiver acceptance emerged as a critical protective factor, particularly salient in school contexts where familial affirmation can counteract peer rejection. However, these protective associations were modest and should be interpreted as partial rather than complete buffers against intersectional victimization. Grounded in MST and TIMSM, these findings emphasize the need for comprehensive, intersectionally informed interventions that span family, school, and digital domains. Although these findings reflect the experiences of U.S. Latine LGBTQ+ adolescents, they underscore the importance of addressing intersecting forms of identity‐based victimization within the social and developmental contexts in which youth live. Promoting affirming environments, empowering caregivers, and addressing online harms are essential steps toward supporting Latine LGBTQ+ youth in the United States in envisioning and pursuing futures rooted not in fear or exclusion but in belonging and hope.

## AUTHOR CONTRIBUTIONS

**Joshua G. Parmenter:** Writing – original draft; writing – review and editing; investigation. **David G. Zelaya:** Writing – original draft; writing – review and editing; methodology; investigation. **Aldo M. Barrita:** Conceptualization; investigation; writing – original draft; methodology; validation; writing – review and editing; formal analysis; data curation; supervision; software; visualization. **Roberto L. Abreu:** Conceptualization; writing – original draft; investigation; methodology; writing – review and editing; formal analysis; data curation; validation; visualization; software; supervision. **Ryan J. Watson:** Funding acquisition; methodology; writing – review and editing; writing – original draft; project administration; investigation; conceptualization. **Nathan Williams:** Writing – review and editing; writing – original draft; investigation.

## FUNDING INFORMATION

This research was supported by the National Institutes of Health K01DA047918.

## CONFLICT OF INTEREST STATEMENT

We have no known conflicts of interest to disclose.

## ETHICS STATEMENT

This study was conducted following the research ethical principles and standards in compliance with the Institutional Review Board of the University of Connecticut, approved on 1/7/22 (H21‐0087).

## CONSENT STATEMENT

Participants were presented with an informed consent online form before participation.

## Supporting information


**Supplemental Table 1.** Descriptive statistics and correlations between online or school‐based intersectional bullying items and key psychosocial outcomes.

## Data Availability

The data that support the findings of this study are available from the corresponding author upon reasonable request.

## References

[jora70247-bib-0001] Abreu, R. L. , Barrita, A. , Sostre, J. P. , Parmenter, J. , & Watson, R. (2025). Interest in *PrEP*, cyberbullying, internalized LGBTQ stigma, and parental support among Latinx sexual and gender diverse youth. Health Psychology, 44(3), 266–274. 10.1037/hea0001455 39992772

[jora70247-bib-0002] Abreu, R. L. , Hernandez, M. , Ramos, I. , Badio, K. S. , & Gonzalez, K. A. (2022). Latinx bi+/plurisexual individuals' disclosure of sexual orientation to family and the role of Latinx cultural values, beliefs, and traditions. Journal of Bisexuality, 23(1), 1–26.

[jora70247-bib-0003] Abreu, R. L. , & Kenny, M. C. (2018). Cyberbullying and LGBTQ youth: A systematic literature review and recommendations for prevention and intervention. Journal of Child & Adolescent Trauma, 11(1), 81–97. 10.1007/s40653-017-0175-7 32318140 PMC7163911

[jora70247-bib-0004] Abreu, R. L. , Lefevor, G. T. , Barrita, A. , Gonzalez, K. A. , & Watson, R. J. (2023). Intersectional microaggressions, depressive symptoms, and the role of LGBTQ‐specific parental support in a sample of Latinx sexual and gender minority youth. Journal of Adolescence, 95, 584–595. 10.1002/jad.12139 36680329

[jora70247-bib-0005] Abreu, R. L. , Skidmore, S. , Barrita, A. , Sostre, J. P. , Lefevor, G. T. , & Watson, R. (2026). Substance use, parental and teacher support, and mental health outcomes among Latinx sexual and gender minority youth. Journal of Homosexuality, 73(4), 983–1004. 10.1080/00918369.2025.2496200 40353813

[jora70247-bib-0006] Angoff, H. D. , & Barnhart, W. R. (2021). Bullying and cyberbullying among LGBQ and heterosexual youth from an intersectional perspective: Findings from the 2017 national youth risk behavior survey. Journal of School Violence, 20(3), 274–286. 10.1080/15388220.2021.1879099

[jora70247-bib-0007] Arcadepani, F. B. , Eskenazi, D. Y. , Fidalgo, T. M. , & Hong, J. S. (2021). An exploration of the link between bullying perpetration and substance use: A review of the literature. Trauma, Violence & Abuse, 22(1), 207–214. 10.1177/1524838019837593 31046605

[jora70247-bib-0008] Barrita, A. , Abreu, R. L. , Carbajal, I. , Parmenter, J. G. , & Watson, R. (2026). *Alcohol and native language*: Alcohol use as a coping strategy for intersectional microaggressions among sexual and gender minoritized Latinx youth. Journal of Psychoactive Drugs, 1–11. 10.1080/02791072.2026.2661582 PMC13215679 42026806

[jora70247-bib-0009] Barrita, A. , Abreu, R. L. , Parmenter, J. , & Watson, R. (2025). Protective effects of online safety and parental acceptance for sexual and gender minority Latinx youth: A quantitative analysis of cyberbullying, psychological distress, and coping with alcohol. LGBT Health, 12(7), 490–498. 10.1089/lgbt.2024.0396 40623828 PMC12629678

[jora70247-bib-0010] Barrita, A. , Carbajal, I. , Abreu, R. L. , Chang, R. , Moreno, O. , Garcini, L. M. , & Wong‐Padoongpatt, G. (2024). *Immigration status microaggressions*: A moderated mediation analysis of cultural stress, fear, internalization, and psychological stress among Latinx and Asian college students. Cultural Diversity & Ethnic Minority Psychology, 30(4), 624–636. 10.1037/cdp0000687 38829332 PMC11792918

[jora70247-bib-0011] Barrita, A. , Hixson, K. , Kachen, A. , Wong‐Padoongpatt, G. , & Krishen, A. (2024). *Centering the margins:* A moderation study examining cisgender privilege among LGBTQ+ BIPoC college students facing intersectional microaggressions. Psychology of Sexual Orientation and Gender Diversity, 11(4), 563–573. 10.1037/sgd0000636

[jora70247-bib-0012] Barrita, A. , Parmenter, J. , Abreu, R. L. , Sostre, J. P. , & Watson, R. (2025). Therapy and parental support for LGBTQ+ Latinx and Black youth: A moderated mediation analysis of internalized stigma, school bullying, and psychological distress. Mental Health Science, 3(1), e70009. 10.1002/mhs2.70009

[jora70247-bib-0013] Barrita, A. , Wong‐Padoongpatt, G. , Chang, R. , Abreu, R. L. , & Krishen, A. (2023). *Internalizing the poison*: A moderated mediation analysis of LGBTQ+ BIPoC college students' experiences with intersectional microaggressions. Scholarship of Teaching and Learning in Psychology, 9(4), 373–391. 10.1037/stl0000375

[jora70247-bib-0014] Brietzke, M. , & Perreira, K. (2017). Stress and coping: Latino youth coming of age in a new Latino destination. Journal of Adolescent Research, 32(4), 407–432. 10.1177/0743558416637915 28626298 PMC5469412

[jora70247-bib-0015] Brooks, V. R. (1981). Minority stress and lesbian women. Lexington Books.

[jora70247-bib-0016] Burger, J. , & Pachankis, J. E. (2024). State of the science: LGBTQ‐affirmative psychotherapy. Behavior Therapy, 55(6), 1318–1334. 39443068 10.1016/j.beth.2024.02.011

[jora70247-bib-0017] Espelage, D. L. , Valido, A. , Hatchel, T. , Ingram, K. M. , Huang, Y. , & Torgal, C. (2019). A literature review of protective factors associated with homophobic bullying and its consequences among children & adolescents. Aggression and Violent Behavior, 45, 98–110. 10.1016/j.avb.2018.07.003

[jora70247-bib-0018] Garcia‐Perez, J. (2020). Lesbian, gay, bisexual, transgender, queer+ Latinx youth mental health disparities: A systematic review. Journal of Gay & Lesbian Social Services, 32(4), 440–478. 10.1080/10538720.2020.1764896

[jora70247-bib-0019] Garcini, L. M. , Barrita, A. , Cadenas, G. A. , Domenech Rodríguez, M. M. , Galvan, T. , Mercado, A. , Moreno, O. , Paris, M. , Rojas Perez, O. F. , Silva, M. , & Venta, A. (2025). A decolonial and liberation lens to social justice research: Upholding promises for diverse, inclusive, and equitable psychological science. American Psychologist, 80(1), 1–14. 10.1037/amp0001255 38127489 PMC11190034

[jora70247-bib-0020] Giano, Z. , O'Neil, A. M. , Stowe, M. , & Hubach, R. D. (2021). Examining profiles of Latinx sexual minority adolescents associated with suicide risk. Journal of Immigrant and Minority Health, 23(3), 452–462. 10.1007/s10903-020-01128-w 33389392

[jora70247-bib-0021] Gower, A. L. , Rider, G. N. , McMorris, B. J. , & Eisenberg, M. E. (2018). Bullying victimization among LGBTQ youth: Current and future directions. Current Sexual Health Reports, 10(4), 246–254. 10.1007/s11930-018-0169-y 31057341 PMC6497454

[jora70247-bib-0022] Hatzenbuehler, M. L. , & Pachankis, J. E. (2016). Stigma and minority stress as social determinants of health among lesbian, gay, bisexual, and transgender youth: Research evidence and clinical implications. Pediatric Clinics, 63(6), 985–997. 27865340 10.1016/j.pcl.2016.07.003

[jora70247-bib-0049] Hayes, A. F. (2012). PROCESS: A versatile computational tool for observed variable mediation, moderation, and conditional process modeling.

[jora70247-bib-0023] Hayes, A. F. , & Rockwood, N. J. (2020). Conditional process analysis: Concepts, computation, and advances in the modeling of the contingencies of mechanisms. American Behavioral Scientist, 64(1), 19–54. 10.1177/0002764219859633

[jora70247-bib-0024] Hirsch, J. K. , Cohn, T. J. , Rowe, C. A. , & Rimmer, S. E. (2017). Minority sexual orientation, gender identity status and suicidal behavior: Serial indirect effects of hope, hopelessness and depressive symptoms. International Journal of Mental Health and Addiction, 15(2), 260–270. 10.1007/s11469-016-9723-x

[jora70247-bib-0025] Jones, L. M. , Mitchell, K. J. , Turner, H. A. , & Ybarra, M. L. (2018). Characteristics of bias‐based harassment incidents reported by a national sample of US adolescents. Journal of Adolescence, 65, 50–60. 10.1016/j.adolescence.2018.02.013 29547771

[jora70247-bib-0026] Kosciw, J. G. , Greytak, E. A. , Zongrone, A. D. , Clark, C. M. , & Truong, N. L. (2018). The 2017 National School Climate Survey: The experiences of lesbian, gay, bisexual, transgender, and queer youth in our nation's schools. GLSEN.

[jora70247-bib-0027] Kroenke, K. , Spitzer, R. L. , Williams, J. B. , & Löwe, B. (2009). An ultra‐brief screening scale for anxiety and depression: The PHQ‐4. Psychosomatics, 50(6), 613–621. 10.1016/S0033-3182(09)70864-3 19996233

[jora70247-bib-0028] Meyer, I. H. (2003). Prejudice, social stress, and mental health in lesbian, gay, and bisexual populations: Conceptual issues and research evidence. Psychological Bulletin, 129(5), 674–697. 10.1037/0033-2909.129.5.674 12956539 PMC2072932

[jora70247-bib-0029] Miller, G. A. , & Chapman, J. P. (2001). Misunderstanding analysis of covariance. Journal of Abnormal Psychology, 110(1), 40–48. 10.1037/0021-843X.110.1.40 11261398

[jora70247-bib-0030] Modecki, K. L. , Minchin, J. , Harbaugh, A. G. , Guerra, N. G. , & Runions, K. C. (2014). Bullying prevalence across contexts: A meta‐analysis measuring cyber and traditional bullying. Journal of Adolescent Health, 55(5), 602–611. 10.1016/j.jadohealth.2014.06.007 25168105

[jora70247-bib-0031] Moe, J. , Robins, L. , Corley, P. , Bumpas, C. , Augustine, B. , & Sparkman‐Key, N. (2025). Hope, multiple minority stress and suicidal behavior in diverse LGBTQ populations: A longitudinal study. Counseling Outcome Research and Evaluation, 17, 1–16. 10.1080/21501378.2025.2542154

[jora70247-bib-0032] Needham, B. L. , & Austin, E. L. (2010). Sexual orientation, parental support, and health during the transition to young adulthood. Journal of Youth and Adolescence, 39(10), 1189–1198. 10.1007/s10964-010-9533-6 20383570

[jora70247-bib-0033] Parmenter, J. G. , & Barrita, A. (2026b). Understanding sexual and gender diverse people of color identity affirmation as a form of resistance: Implications for a resistance–resilience model. American Psychologist. Advance online publication. 10.1037/amp0001671 41785133

[jora70247-bib-0034] Parmenter, J. G. , & Barrita, A. (2026a). A preliminary model of intersectional minority stress among sexual and gender diverse black, indigenous, and people of color. Psychology of Sexual Orientation and Gender Diversity, 13(2), 207–217. 10.1037/sgd0000748

[jora70247-bib-0035] Pollitt, A. M. , Fish, J. N. , & Watson, R. J. (2023). Measurement equivalence of family acceptance/rejection among sexual and gender minority youth by disclosure status. Journal of Family Psychology, 37(2), 195–202. 10.1037/fam0001056 36634006 PMC9928906

[jora70247-bib-0036] Puhl, R. M. , Peterson, J. L. , & Luedicke, J. (2012). Strategies to address weight‐based victimization: Youths' preferred support interventions from classmates, teachers, and parents. Journal of Youth and Adolescence, 42(3), 315–327. 23117953 10.1007/s10964-012-9849-5

[jora70247-bib-0037] Purdie‐Vaughns, V. , & Eibach, R. P. (2008). Intersectional invisibility: The distinctive advantages and disadvantages of multiple subordinate‐group identities. Sex Roles, 59(5), 377–391. 10.1007/s11199-008-9424-4

[jora70247-bib-0038] Rivas‐Koehl, M. , Rivas‐Koehl, D. , & McNeil Smith, S. (2023). The temporal intersectional minority stress model: Reimagining minority stress theory. Journal of Family Theory & Review, 15(4), 706–726. 10.1111/jftr.12529

[jora70247-bib-0039] Rosales, R. , Zelaya, D. G. , Moreno, O. , Figuereo, V. , Chavez, S. J. , Ordoñez, S. , Costas, I. , Ponce, M. , & Miranda, R., Jr. (2023). Latinx sexual minority adolescent substance use: State of the science and call for intersectional minority stressors and protective factors. Current Addiction Reports, 10(3), 396–411. 10.1007/s40429-023-00503-5 38774111 PMC11104555

[jora70247-bib-0040] Ryan, C. , Russell, S. T. , Huebner, D. , Diaz, R. , & Sanchez, J. (2010). Family acceptance in adolescence and the health of LGBT young adults. Journal of Child and Adolescent Psychiatric Nursing: Official Publication of the Association of Child and Adolescent Psychiatric Nurses, Inc, 23(4), 205–213. 10.1111/j.1744-6171.2010.00246.x 21073595

[jora70247-bib-0041] Scanlon, F. , Remch, M. , Scheidell, J. D. , Brewer, R. , Dyer, T. V. , Albis‐Burdige, B. , Irvine, N. , Turpin, R. , Parker, S. , Cleland, C. M. , Hucks‐Ortiz, C. , Gaydos, C. A. , Mayer, K. H. , & Khan, M. R. (2024). Posttraumatic stress disorder symptoms and incarceration: The impact on sexual risk‐taking, sexually transmitted infections, and depression among Black sexual minority men in HIV Prevention Trials Network (HPTN) 061. Psychology of Men & Masculinity, 25(1), 44. 38854997 10.1037/men0000458 PMC11156418

[jora70247-bib-0042] Schnarrs, P. W. , Stone, A. L. , Salcido, R., Jr. , Baldwin, A. , Georgiou, C. , & Nemeroff, C. B. (2019). Differences in adverse childhood experiences (ACEs) and quality of physical and mental health between transgender and cisgender sexual minorities. Journal of Psychiatric Research, 119, 1–6. 10.1016/j.jpsychires.2019.09.001 31518909 PMC10675992

[jora70247-bib-0043] Shidlo, A. (1994). Internalized homophobia: Conceptual and empirical issues in measurement. In Lesbian and gay psychology: Theory, research, and clinical applications (pp. 176–205). Sage Publications, Inc. 10.4135/9781483326757.n10

[jora70247-bib-0044] Toomey, R. B. , Shramko, M. , Flores, M. , & Anhalt, K. (2018). Family socialization for racism and heterosexism: Experiences of Latinx sexual minority adolescents and young adults. Journal of Family Issues, 39(13), 3586–3611. 10.1177/0192513X18783807 40881672 PMC12382482

[jora70247-bib-0045] Velez, B. L. , Moradi, B. , & DeBlaere, C. (2015). Multiple oppressions and the mental health of sexual minority Latina/o individuals. The Counseling Psychologist, 43(1), 7–38. 10.1177/0011000014542836

[jora70247-bib-0046] Watson, R. J. , Caba, A. E. , Lawrence, S. E. , Renley, B. M. , McCauley, P. S. , Wheldon, C. W. , Eaton, L. A. , Russell, S. T. , & Eisenberg, M. E. (2024). Examining mental health and bullying concerns at the intersection of sexuality, gender, race, and ethnicity among a national sample of sexual and gender diverse youth. LGBT Health, 11(1), 20–27. 10.1089/lgbt.2023.0072 37668602 PMC11074444

[jora70247-bib-0047] Zhang, S. , Liu, M. , Li, Y. , & Chung, J. E. (2021). Teens' social media engagement during the COVID‐19 pandemic: A time series examination of posting and emotion on Reddit. International Journal of Environmental Research and Public Health, 18(19), 10079. 10.3390/ijerph181910079 34639381 PMC8507823

[jora70247-bib-0048] Zongrone, A. D. , Truong, N. L. , & Kosciw, J. G. (2020). Erasure and resilience: The experiences of LGBTQ students of color, Latinx LGBTQ youth in U.S. schools. GLSEN.

