# Supplementary material for: An intersectional approach for the effects of school bullying & cyberbullying on beliefs about the future among Latine LGBTQ+ youth in the United States
Source: J Res Adolesc. 2026 Aug 10;36(3):e70247. doi: 10.1111/jora.70247 (PMC13454855; doi:10.1111/jora.70247)
Supplement: Supplementary file 1 [file JORA-36-0-s001.docx]

**Supplemental Table 1**

*Descriptive Statistics and Correlations Between Online or School-Based Intersectional Bullying Items and Key Psychosocial Outcomes*

| **Type of Bullying Item** | **M (SD)** | **LGBTQ+ Internalized Stigma** | **Depression & Anxiety** | **Beliefs About the Future** | **Caregiver LGBTQ+** |
| --- | --- | --- | --- | --- | --- |
| **Online** |  |  |  |  |  |
| Gender | 1.84 (1.15) | .159*** | .264*** | -.202*** | -.216*** |
| Race/Ethnicity | 1.64 (1.05) | .156*** | .192*** | -.168*** | -.175*** |
| Sexual Identity | 2.02 (1.23) | .152*** | .244*** | -.172*** | -.194*** |
| Immigrant Identity | 1.18 (0.63) | .112*** | .165** | -.160** | -.120*** |
| Masculinity/Femininity | 1.80 (1.14) | .161*** | .231*** | -.155*** | -.216*** |
| Weight | 1.54 (1.00) | .159*** | .194*** | -.128*** | -.180*** |
| Disability | 1.36 (0.87) | .137*** | .205*** | -.103*** | -.144*** |
| Religion | 1.30 (0.79) | .132*** | .117*** | -.130*** | -.169*** |
| **School-Based** |  |  |  |  |  |
| Gender | 1.85 (1.16) | .190*** | .294*** | -.222*** | -.293*** |
| Race/Ethnicity | 1.76 (1.07) | .161*** | .203*** | -.162*** | -.186*** |
| Sexual Identity | 2.09 (1.23) | .153*** | .265*** | -.122*** | -.233*** |
| Immigrant Identity | 1.21 (0.68) | .136*** | .165*** | -.183*** | -.123*** |
| Masculinity/Femininity | 2.11 (1.21) | .188*** | .252*** | -.146*** | -.247*** |
| Weight | 1.73 (1.04) | .132*** | .211*** | -.108*** | -.175*** |
| Disability | 1.41 (0.90) | .126*** | .187*** | -.192*** | -.166*** |
| Religion | 1.30 (0.79) | .129*** | .187*** | -.186*** | -.173*** |

Note: ^**^*p* < .01. ^***^*p* < .001.
